# Nitrogen doping to atomically match reaction sites in microbial fuel cells

**DOI:** 10.1038/s42004-020-0316-z

**Published:** 2020-06-01

**Authors:** Xiaoshuai Wu, Yan Qiao, Chunxian Guo, Zhuanzhuan Shi, Chang Ming Li

**Affiliations:** 1 Institute of Materials Science and Devices, Suzhou University of Science and Technology, Suzhou, 215011 China; 2 Institute for Clean Energy and Advanced Materials, School of Materials and Energy, Southwest University, Chongqing, 400715 China; 3 Chongqing Key Laboratory for Advanced Materials and Technologies of Clean Energies, Chongqing, 400715 P. R. China; 4 Institute for Advanced Cross-field Science and College of Life Science, Qingdao University, Qingdao, 266071 P. R. China

**Keywords:** Biocatalysis, Fuel cells, Electrochemistry

## Abstract

Direct electron transfer at microbial anodes offers high energy conversion efficiency but relies on low concentrations of redox centers on bacterium membranes resulting in low power density. Here a heat-treatment is used to delicately tune nitrogen-doping for atomic matching with Flavin (a diffusive mediator) reaction sites resulting in strong adsorption and conversion of diffusive mediators to anchored redox centers. This impregnates highly concentrated fixed redox centers in the microbes-loaded biofilm electrode. This atomic matching enables short electron transfer pathways resulting in fast, direct electrochemistry as shown in *Shewanella putrefaciens* (*S. putrefaciens*) based microbial fuel cells (MFCs), showing a maximum power output higher than the conventional non-matched nitrogen-doped anode based MFCs by 21 times. This work sheds a light on diffusion mediation for fast direct electrochemistry, while holding promise for efficient and high power MFCs.

## Abstract (web summary)

In microbial fuel cells direct electron transfer offers high energy conversion efficiency, but low concentrations of redox centers on bacterial membranes result in low power density. Here nitrogen-doping is fine tuned to match Flavin reaction sites, converting diffusive mediators to anchored redox centers toward direct electrochemistry.

## Introduction

As a renewable energy source to harvest electricity by oxidizing substrates (electron donor) such as organic wastes, microbial fuel cells (MFCs) have received great attention in recent years^1,2^. The practical application of MFCs is still restrained by its relatively low power density in comparison to the conventional H_2_-O_2_ fuel cells, due to the poor electron transfer between bacteria and electrode^3–8^. The electron transfer pathways of MFCs anodes are generally divided into two processes, namely, direct electron transfer and diffusive redox species-mediating transfer. It has also been noted that the electron transfer mediated by endogenously generated electron shuttles from bacteria is even more advantageous in MFCs than the membrane active centers-enabled direct electron transfer, since such highly concentrated mediators can produce high current density while eliminating adding additional electron mediators^9–12^. As an important redox moiety for electron transfer cycles in MFCs, *Shewanella sp*. as a model organism has been widely used and intensively investigated in MFCs. Flavin^13,14^ as an electron mediator permits bacteria to utilize a remote electron acceptor that was is accessible to the cells. Flavin mononucleotide (FMN) has been utilized as a two-electron/proton redox mediator as FMN + 2e^−^ + 2 H^+^ → FMNH_2_^9,15^, and the electron transfer mediated by Flavin is executed at 1, 5-nitrogens via a two-electron reaction between their Quinone and hydroquinone species^11^. FMN can be either in free-state or bonded state in MFCs anode. It has been proposed a possibility that the concentration of free Flavin in MFCs anode could greatly affect the power output while the bonded Flavins could allow direct electron transfer between the outer membrane cytochromes and the electrode^16,17^, but it has not been proved. The direct electrochemistry (DEC) is always more efficient and faster than a diffusive redox species-mediated indirect electrochemistry in the MFCs anode due to its short electron pathways but it is still very challenging to achieve high current density due to lack of high density of reaction active centers for the fast direct electron transfer. Currently, MFCs anodes mainly rely on mediation chemistry, an indirectly electron mediator-based electron transfer process^6,9,13^, in which the lowly diffusive flux of electron mediators as electron shuttles often suffers from diffusion limit presenting a diffusion control process. In particular, the MFC anodes are porous and the diffusion limiting even more seriously affect the electricity harvest in MFCs leading to high energy losses^16^. There is very high motivation to develop efficient approaches to accelerate the direct electrochemistry of mediators such as Flavin for high power MFCs while exploring fundamental insights of the direct electrochemistry.

Functional nanomaterials have been used to modify MFCs anodes to improve the electron transfer process by either overcoming the steric effect of diffusive redox shuttles such as Flavin to access the electrode surface or offering proximity of electrode nanomaterial to cell membrane for direct electrochemistry^11^. However, these approaches can still not significantly improve the power density of MFCs. Among various functional materials used in MFCs, nitrogen-doped carbon materials have been investigated due to their good electrical conductivity, rich surface functional groups as well as unique electronic structure^18–20^. As an example, nitrogen-doped carbon nanoparticles have been used to increase anodic adsorption of flavins for facilitating shuttle-mediated extracellular electron transfer^21^. Previous work has applied plasma to implant N^+^ ion for MFCs anode modification for enhanced biofilm growth and improved interaction between bacteria and electrode^22^. Another work has demonstrated that the use of amine-terminated ionic liquid (IL-NH_2_) functionalized carbon nanotubes (CNTs) could improve the Flavin based interfacial electron transfer^23^. Although nitrogen-doped carbon materials as electrode materials have shown improved performance of MFCs, the doping process has never been rationally tailored to match the reaction sites of FMN for highly concentrated fixed reaction centers and further to convert a mediating electron transfer pathway to a direct electrochemistry process.

In this work, a thermal treatment is used to delicately control nitrogen doping for atomic matching with Flavin (mediator) reaction sites, which results in strong adsorption to convert diffusive mediator molecules for anchored redox centers. The followed microbes loading on the electrode surface with completely fixed highly concentrated redox centers for a short electron pathway to eventually convert mediating electron transfer to a fast direct electrochemistry. The rational nitrogen-matching process is investigated in detail. Further, the detail atomic matching catalytic mechanism for a fast direct electrochemistry is discussed. Application of such a direct electrochemistry behavior of FMN enabled by a rationally doped nitrogen for matching atomic reaction sites is further demonstrated, and significant improvement has been achieved.

## Results

### Material characterizations

**Fig. 1:**
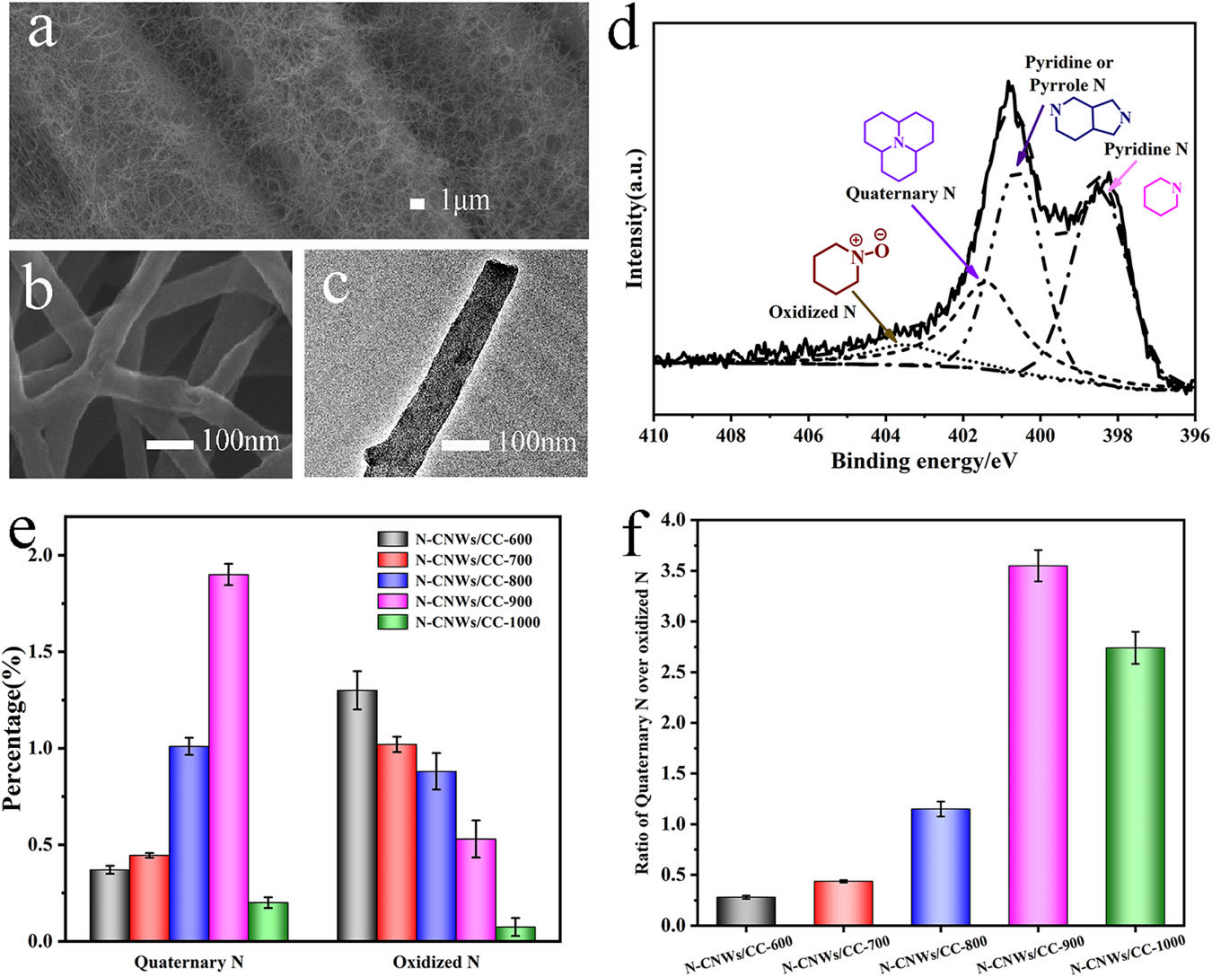
Characterization of rationally nitrogen-doped carbon nanowire electrodes. SEM image (**a**), high magnification SEM image (**b**) and TEM image (**c**) of N-CNWs/CC-900. **d** XPS N1s spectrum of N-CNWs. **e** The percentage histogram of quaternary N and oxidized N of N-CNWs samples prepared at different temperatures. **f** The ratio histogram of Quaternary N over oxidized N of N-CNWs samples prepared at different temperatures. Error bars represent one standard deviation.

Structure and morphology of the nitrogen-doped carbon nanowires (N-CNWs) were investigated by SEM and TEM. SEM image in Fig. 1a show a uniform distribution of randomly oriented carbonized nanowires and the network structure like a “fishing nets” coated on the surface of carbon cloth fibers. The morphologies of N-CNWs similar with that of the PANI nanowires before carbonization (Supplementary Fig. 1a). The high-magnification FESEM image (Fig. 1b) and TEM image (Fig. 1c) illustrated the wire-like structures of N-CNWs with 60–100 nm in diameter and several microns in length. More interestingly, the results show that the carbonization temperature has no obvious effect on the morphology, pore structure, surface wettability and electron conductivity of the nanowires after carbonized at different temperatures, which are shown in Supplementary Fig. 1b–d and Supplementary Table 1. Apparently, the change of the carbonization temperature mainly affects the doping amount of the nitrogen atoms on the surface of the nanowires and the doping sites, which reduces the other variables in the process of studying the direct electrochemical effects of nitrogen atoms on the electron mediator and contributes to the accurate localization of the regularity and the molecular modeling of its surface. Furthermore, the representatives Raman spectra (Supplementary Fig. 1e) of N-CNWs reveal that the 2D (G’) band at 2700 cm^−1^ can be observed clearly as the increase of the carbonization temperature. The enhanced intensity of the 2D band suggests an increase of the ratio of graphitic-like structures.

The surface properties of the N-CNWs were characterized with XPS. The XPS survey spectra (Supplementary Fig. 2) show the presence of C (BE ≈ 284.5 eV), N (BE ≈ 400 eV), and O (BE ≈ 531.5 eV) atoms on the surface, which shows that for N atom, the N1s peak of N-CNWs (Supplementary Fig. 3b–g) is different from plain PANI nanowire without carbonization (Supplementary Fig. 3a), and is well deconvoluted^24–26^ into four peaks located at 398.47, 400.62, 401.4, and 403.5 eV attributing to pyridine N (N-6), pyridine or pyrrole N (N-5), quaternary N (N-Q) and oxidized N (N-X), respectively. Distribution of nitrogen species obtained from the deconvolution of the N1s peak indicates different N-doping hexagonal carbon structures for different N-CNWs. As show in Supplementary Table 2, for N-CNWs/CC-600, N-CNWs/CC-700, N-CNWs/CC-800, and N-CNWs/CC-900 electrode, the change of N-6 and N-5 is not obviously. The most obvious change is the distribution of N-Q and N-X types (Fig. 1e, f), and the ratios of these two types nitrogen atoms are 0.28, 0.437, 1.15 and 3.55, respectively. Although the N-CNWs/CC-1000 electrode has a significant decrease in the content of its nitrogen atoms at the high carbonization temperature, the ratio of N-Q and N-X has reached 2.74, which is relatively less than N-CNWs/CC-900 electrode but still greater than the ratios of other three N-CNWs/CC electrodes. It is noted that the noise to the measured N1s peak in XPS spectra increases with the carbonization temperature. It is very likely to be caused by the sharply decreased amount of N atoms (the doping amount of N actually is not large), but the results can be used for quantitative analysis. Incorporation of heteroatoms into carbon could tailor its electron-donor properties for required electrical and chemical performance. In this case, the different N-doping hexagonal carbon structures will influence the interaction between heterocyclic structure of the soluble electron mediator (secreted by electroactive bacteria) and material surface, which could lead to a higher mediator retention at the interface between the electrode and biofilm^27–29^.

### Converting diffusive mediators to anchored DEC reaction centers

**Fig. 2:**
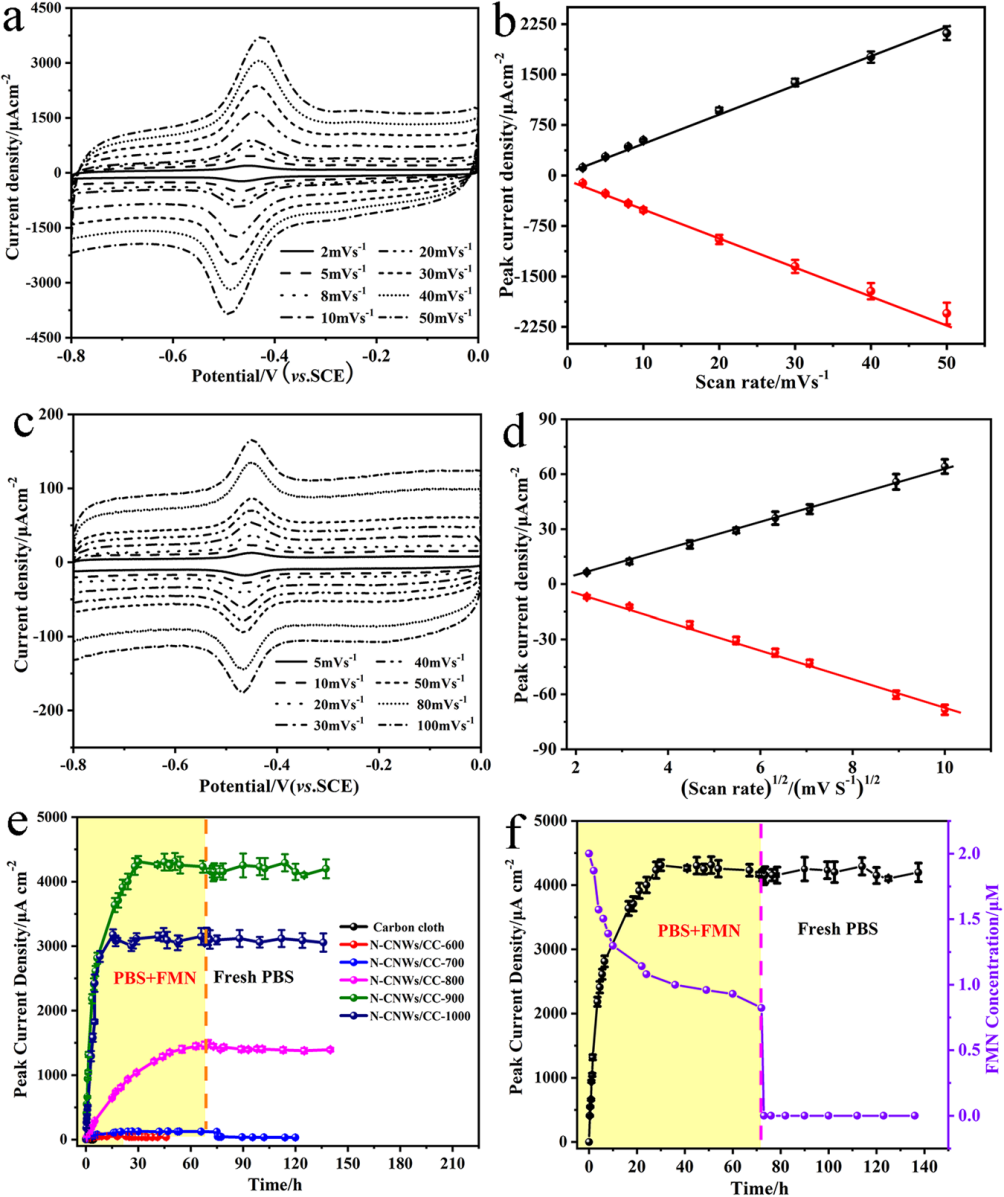
Flavin adsorption characteristics on different N-doped electrodes and corresponding electrochemical behaviors. Peak current density and CV curves at different scan rate of the N-CNWs/CC-900 electrode (**a**, **b**) and the plain carbon electrode (**c**, **d**) measured in 2 μM FMN. **e** Peak current density against time of N-CNWs/CC in 2 μM FMN solution with 0.1 M phosphate buffer (PBS) at PH = 7.4. **f** Peak current density and FMN con-centration against time of the N-CNWs/CC-900 electrode. Error bars represent one standard deviation.

CVs of differently N-doped CNWs/CC electrodes by well-tuned thermal processes were measured in 2 μM FMN and 50 mM potassium ferricyanide solution (Supplementary Fig. 4). The results show that the electroactive surface areas of different electrodes after carbonizations at different temperatures are indeed different but are not significant (Supplementary Fig. 4a). After normalized the electrode surface areas at different carbonization temperatures, the peak current of different electrode measured in 2 μM FMN increases with the increased heat-treated temperature (Supplementary Fig. 4b–d) until reaching the maximum one at N-CNWs/CC-900 electrode, then drops with the further increased heat-treated temperature. Further, the relation of both the ratio of N-Q and N-X determined from the XPS data for these electrodes (Supplementary Fig. 2) and the peak current versus the heat-treated temperature in Supplementary Fig. 5 illustrates that the peak current is proportional to the ratio of N-Q and N-X and N-CNWs/CC-900 electrode delivers the highest peak current. CVs of N-CNWs/CC-900 and conventional non-matched nitrogen doped plain carbon cloth electrodes were measured at different scan rates. Interestingly, the relation of the peak current versus the scan rate for N-CNWs/CC-900 is linear (*R*^2^ = 0.996) (Fig. 2a, b), indicating a surface reaction controlled process without diffusion limit. In contrast, the anodic peak current of conventional non-matched nitrogen doped plain carbon cloth electrode versus square root of scan rate shows a linear relationship (*R*^2^ = 0.995) (Fig. 2c, d), suggesting that the electrochemical reaction on carbon cloth surface is subject to a diffusion controlled process. Subsequently, the surface properties of the N-CNWs/CC-900 electrode after FMN adsorption was characterized with XPS as shown in Supplementary Fig. 6, in which the peak located at 132 eV attributing to phosphate, indicating that FMN could anchor on the surface of electrode. This result clearly confirms that the N-doped carbons with 900 °C carbonization have anchored FMN on the electrode surface as fixed redox centers to enable surface-controlled redox reactions.

To examine whether FMN can be stably anchored on the electrode surface as the reaction centers, CVs of different N-CNWs/CC electrodes were measured in FMN phosphate buffer solution for 60 h, and then placed these FMN-immobilized electrodes in fresh plain phosphate buffer solution to measure their CVs again. The peak current of CVs can be used to measure the FMN adsorption ability and the time-course anodic peak current for the various N-CNWs/CC electrodes as shown in Fig. 2e. It is observed that as the increase of the carbonization temperature, the FMN adsorption ability of the N-CNWs greatly increases until 900 degree and then decrease with the temperature increasing (FMN_CNWs/CC_ < FMN_N-CNWs/CC-600_ < FMN_N-CNWs/CC-700_ < FMN_N-CNWs/CC-800_ < FMN_N-CNWs/CC-1000_ < FMN_N-CNWs/CC-900_). Obviously, the N-CNWs/CC-900 electrode has the highest FMN adsorption while the plain CNWs/CC electrode without nitrogen doping exhibits the least adsorption of FMN. More importantly, when placing these FMN immobilized electrodes in plain phosphate buffer solution, the stabilities of FMN immobilizations for different electrodes are different. Supplementary Fig. 7 shows that all FMN-immobilized CNWs/CC including the N-CNWs/CC electrodes heat-treated at temperatures <800 °C display fast desorption of FMN; nevertheless, when carbonizing the electrodes higher than 800 °C (Fig. 2e), the FMN immobilizations are quite stable. In particular, the N-CNWs/CC-900 electrode exhibits the most stable anchored FMN adsorption with insignificant desorption even after immersing in fresh phosphate buffer after 60 h. When using CVs to characterize the adsorption/desorption process on the electrodes for stability of FMN immobilization, UV spectrophotometer was used to monitor the changes of FMN in solutions for the CV time-course measurements. Obviously, with FMN adsorption increase on the electrode, the CV peak current increases and the FMN concentration in solution should be correspondingly decreased. Figure 2f shows the UV results measured for the N-CNWs/CC-900 electrode, which is in very good agreement with the CV results, the amount of Flavin anchored on the surface of the N-CNWs/CC-900 electrode estimated from the measured change of FMN in solution by UV spectrophotometer is ~1.1776 μM.

**Fig. 3:**
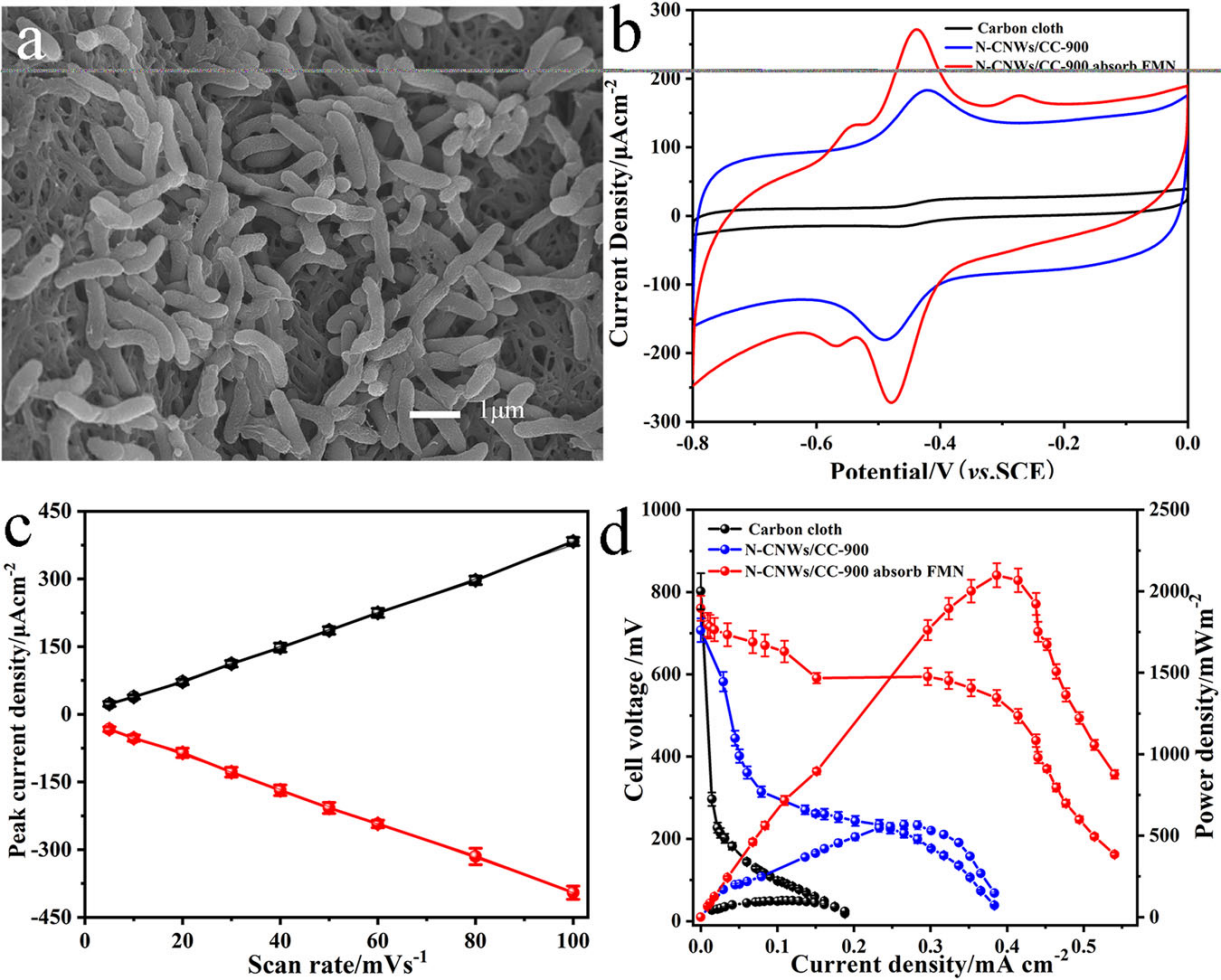
Performance enhancement by atomically matching the reaction sites of FMN. **a** SEM micrographs of *S. putrefaciens* cells adhered on the surface of FMN-immobilized electrode. **b** CVs of FMN-immobilized electrode, N-CNWs-900 and plain carbon anode *S. putrefaciens* CN32 suspension with 18 mmol L^−1^ lactate medium. **c** Peak current density against scan rate of FMN-immobilized electrode measured in an anaerobic of *S. putrefaciens* CN32 suspension with 18 mmol L^−1^ lactate medium. **d** The comparison of the polarization curve and the power output of the MFCs with plain carbon anode (black line), N-CNWs/CC-900 (blue line) and FMN-immobilized electrode (red line). Error bars represent one standard deviation.

To examine the performance enhanced by the reaction sites-matched FMN, microbes was firstly loaded on the FMN-immobilized electrode. The surface morphology of the biofilm was examined with FESEM (Supplementary Fig. 8), a large amount of *S. putrefaciens* cells accumulate on the electrode surface, the graphs with high magnification (Fig. 3a) clearly illustrate that the cells on the electrode surface and cross-link each other to form a network. Obviously, the cell adhesion on the electrode surface for a superior biofilm could promote the bacteria have a suitable microenvironment for extracellular electron transfer during the electrochemical reaction, further proving the atomically matched reaction sites with microbes could obviously also promote the growth of the biofilm catalyst. It is worth to note that the flavins can obtain electrons from the bacteria cells through the outer membrane bounded flavodoxins without entering the periplasmic space of the cells. The electro catalytic analyses of FMN-immobilized electrode, N-CNWs-900 and plain carbon anode were investigated in *S. putrefaciens* CN32 MFCs (Fig. 3b). The well-defined redox peak centered at about −0.46 V vs. SCE can be assigned to the electrochemical behavior of flavins and the redox peak appeared at −0.56 V vs. SCE should be attributed to the Flavin adsorption on the anode surface, which well agrees with the reported redox peaks^30–32^. The relation of the peak current versus the scan rate for FMN-immobilized electrode in an anaerobic of *S. putrefaciens* CN32 suspension with 18 mmol L^−1^ lactate medium is linear (*R*^2^ = 0.999) (Fig. 3c and Supplementary Fig. 9), indicating a surface reaction controlled process without diffusion limit. In contrast, the anodic peak current of conventional non-matched nitrogen doped plain carbon cloth electrode versus square root of scan rate shows a linear relationship (*R*^2^ = 0.997) (Supplementary Fig. 10), suggesting that the electrochemical reaction on carbon cloth surface is subject to a diffusion controlled process. This result clearly confirms that atomic matching can convert diffusive mediator pathway to a direct electrochemistry. Then, the power density performance of the rationally reaction sites-matched anode was studied in *S. putrefaciens* CN32 MFCs. The maximum power density of the atomic matched nitrogen doped anode can be enhanced to 2102 mW m^−2^, which is around 21-fold higher than that of the conventional non-matched nitrogen doped MFC anode (Fig. 3d). In addition, the internal resistance of the MFCs corresponding to its maximum power output is ~1404 Ω^33^. More importantly, the performance of the atomic matched nitrogen doped anode based MFCs is distinctly superior to the reported MFCs as in Supplementary Table 3 that use similar MFCs setups, bacterial strains similar to *Shewanella* species (one culture) and operating conditions. It is worthy of a note that MFCs with mixed culture could offer high power density due to the synergistic effect. Our lab plans to investigate this type of MFCs setup while applying this atomic matching adsorption concept to convert mediation pathways to direct electrochemistry for further exploration of both theoretical and practical aspects. The reason could be that the large amount of adsorbed flavins on the N-CNWs/CC-9000 interface, which establish an electron transfer channel connecting the bacteria to the electrode interface and guarantee a fast direct electrochemistry. In this case, the anchored FMN and loading amount of the bacteria biofilm on the anode is crucial for the flavins based EET.

## Discussion

**Fig. 4:**
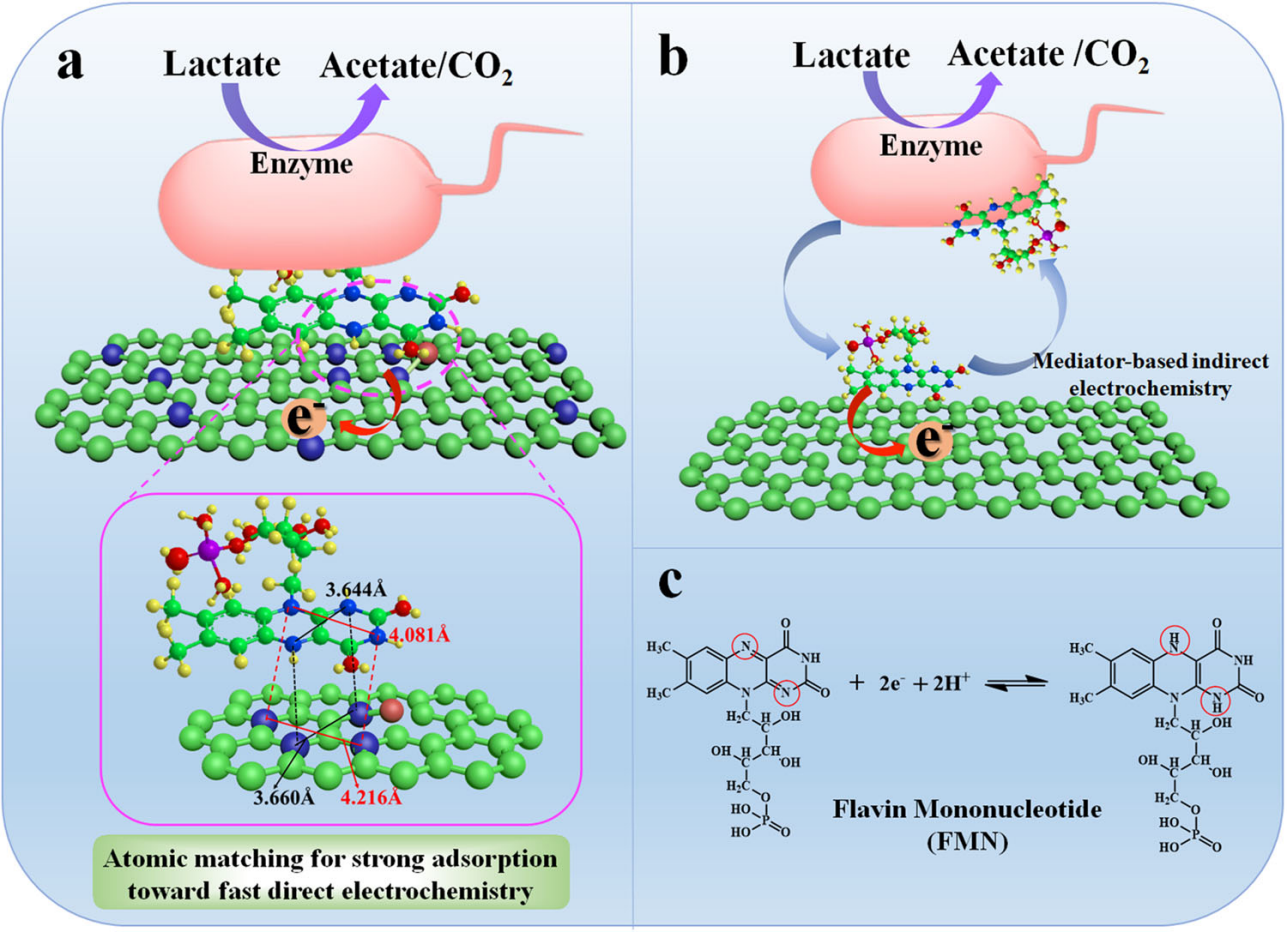
Schematic illustration of atomic matching catalysis mechanism. Schematic illustration of the enhancement mechanism of N-doped carbon structure for the direct electrochemistry of FMN. **a** Molecular structures and atomic distance of FMN and nitrogen sites of N-CNWs as well as the interaction between FMN and N-CNWs. **b** Schematic illustration of mediator-based indirect electrochemistry between FMN and non-matched nitrogen doped electrode. **c** Two-electron redox reaction equation of FMN and its chemical molecular structure. The two nitrogen sites highlighted by red circles is the active sites during the redox reaction.

Considering the concentration of self-excreted electron shuttles are often found at nM or μM concentrations^34,35^, while many other ions (e.g. potassium, sodium, phosphate, chloride, lactate and bicarbonate) are present in mM concentrations, the transport of electron shuttles by migration is negligible^16^. Thus, the total current density obtained by electroactive bacteria using electron shuttles is limited by the diffusion of electron mediator. More importantly, the relatively small diffusion coefficients of organic mediator molecules indicate that the diffusion is an inherently slow process especially in a porous anode due to the very low concentration gradient and extremely narrow mass transport channels. Previous works have demonstrated that electron transfer between carbon electrodes and flavins is also relatively sluggish and surfaces designed to better interact with these compounds would deliver a higher electron transfer rate. It has been reported that modification of an electrode surface through nitrogen doping enhances electron transfer between electrode and microbe and proposes that it may due to enhanced adsorption ability of electron shuttles adsorption^27^. However, fundamentally the mechanism of the interaction between the electron shuttles and electrode surface as well as its effect on direct electrochemistry has not been systematically investigated yet. The nitrogen doping effect on the interaction of nitrogen and FMN mediator was first studied by us. Figure 4c shows that the FMN-mediated electron transfer occurs at 1,5-nitrogens via a two-electron redox reaction between quinone (FMN) and hydroquinone (the oxidation product of FMN) species^11,36^. It is noted that the steric distance between the two electroactive sites (1,5-nitrogens) is 3.644 and 4.081 Å between the other two nitrogen (2,4-nitrogens) of FMN at the lowest energy state (Fig. 4a). Interestingly, our measured XPS and corresponding simulation results clearly shows when the ratio of these N-Q and N-X for the nitrogen doping is three, the nitrogen distribution forms such a structure that the steric distance between N-X and N-Q is 3.660 and 4.216 Å between other two N-Q, respectively, which generates an atomic match with electroactive sites of FMN mediator. We argue that FMN can have strong interactions to preferentially adsorb on the surface with these atomically matching reaction sites and even anchor these FMN mediators on electrode surface for high adsorption of FMN. Further, it is known that FMN is a normally endogenously generated electron shuttle from bacteria, which can be easily pass through the membrane and thus should possess superior affinity to the cell membrane. We can reasonably argue that although the Flavin is just anchored on the surface of electrode, it could strongly attract the membrane of the microbe strains by the extremely high affinity to interact or adsorb or offer the most proximity to the cell membrane with very short electron transfer paths during the bacteria impregnation process for direct electrochemistry without the diffusion limit^11^. In addition, the atomic matched 2-electroactive centers of FMN with nitrogen on the electrode can allow simultaneous fast direct two-electron transfer process. This is why the highly concentrated, atomically matched FMN anode can enhance more than 21 folds high power density. This eventually convert a mediating electron transfer process to an anchored redox center (mediator)-based direct electrochemistry. Figure 4 schematically shows this atomic matching catalysis mechanism to convert a mediating electron transfer process (Fig. 4b) to a fast direct electrochemistry electron pathway (Fig. 4a), which gives vivid schematic explanation why the doping ratio of 3 to 1(N-Q and N-X) nitrogen can deliver the highest electrocatalytic activity and the largest catalytic current.

Notably, the carbonization temperature of PANI can be used to delicately tune the doping ratio of nitrogen on the electrode. There is optimal heating temperature (900 °C) to achieve a doping ratio of nitrogen (N-Q and N-X) for 3:1. The temperatures lower than 900 °C cannot produce enough N-Q to reach a ratio of 3:1, while a carbonization temperature higher than 900 °C may burn out carbon too much resulting in a less total nitrogen doping and also a lower doping ratio. The mechanism of the interaction between the electrode surface and electron shuttles is modeled via an appropriate theoretical approach. The calculations were based on spin-polarized density functional theory (DFT) using projector augmented wave (PAW) methods, as implemented in the Vienna ab initial simulation package (VASP). As shown in Supplementary Fig. 11, a plane-wave basis set with a kinetic-energy cut-off of 400 eV was used to expand the wave function of valence electrons. The adsorption energy of FMN adsorbed on different electrodes interface were shown in Supplementary Table 4, the *E*_ads_ of FMN adsorbed on N-CNWs/CC-900 (−0.260 eV) surface was smaller than the carbon surface (−0.007 eV), N-CNWs/CC-800 (−0.088 eV) and N-CNWs/CC-700 (−0.059 eV). Clearly, the results indicate that the surface of N-CNWs/CC-900 by the atomic match with the reaction sites of FMN was more favorable for Flavin adsorption, which is in good agreement with our experimental results discussed above.

DFT calculation for N-CNWs/CC-1000 was also performed. Interestingly, the ratio of Quaternary N and Oxidized N of N-CNWs/CC-1000 is similar with N-CNWs/CC-900 according to the XPS configuration results of N-doping (Supplementary Table 2), the modeling and theoretical calculation results is also the same, indicating both can enable the atomic match. However, the matched atomic active sites after 1000 °C carbonization should be less than 900 °C carbonization due to its lower electrocalytic activity even with a same loss ratio of Quaternary to Oxidized N indicated by XPS results. The measured electrochemical active surface area of the former is smaller than the latter, which strongly supports the discussion above.

To further confirm whether the atomic matching catalysis mechanism has a universal significance, the electrochemical behaviors of different electrode in phosphate buffer containing 2 μM riboflavin (RF) were investigated. RF is another Flavin mediator secreted from *Shewanella spp*, and its electroactive sites are similar with FMN (Fig. 4c and Supplementary Fig. 12a). Supplementary Fig. 10b–d illustrate that the N-CNWs/CC-900 electrode also has the highest redox peak current and the lowest interfacial charge transfer resistance in RF solution. Obviously, the atomic matching catalysis enhancement mechanism of FMN is also applicable for RF.

In conclusion, a thermal treatment is used to delicately tune nitrogen doping for atomic matching with the diffusive Flavin (mediator) reaction sites resulting to convert diffusive mediator molecules for anchored redox centers, and the followed microbes loading on the electrode surface with fully fixed atomic matched redox centers create an environment for a short electron pathway leading to fast direct electrochemistry. This eventually converts mediating electron transfer pathway to a fast direct electrochemistry by an atomic matching mechanism. The superior atomic match not only provides strong adsorption of flavins as fixed reaction centers but also enabling simultaneously 2-sites direct electrochemistry pathway for extremely higher power density, delivering a maximum power output of 2102 mW m^−2^, which is more than 21 times higher than that of conventional non-matched nitrogen doped MFC anode. Considering the facile synthetic process, the long-term stability in nanostructures and surface properties and the superior electro catalytic performance of the N-CNWs/CC-900, this work demonstrates a feasible approach to turns a diffusive redox species-mediated electron transfer into a direct electrochemistry process, while exploring a new fundamental insights of direct electrochemistry, which can go through an atomic matching mechanism to realize fast and efficient direct electrochemistry.

## Materials

### Nitrogen doped nanowires synthesis and characterizations

All the nitrogen-doping nanowires were synthesized by carbonized PANI nanowires that deposited on carbon cloth surface. To prepare PANI nanowires by electrochemical deposition procedure: (1) all carbon cloth substrates were cleaned in 0.1 mol L^−1^ H_2_SO_4_ solution prior to deposition. (2) Placing carbon cloth in 1 M HClO_4_ solution containing 0.3 M aniline. (3) Polymerization was conducted with a constant current deposition with a current density of 5 mA cm^−2^ for 3 min, and then a current density of 2.5 mA cm^−2^ for 10 min. (4) after polymerization, the electrode was washed in DI water carefully and dried at room temperature. Finally, the PANI modified carbon cloth carbonized at 600 °C, 700 °C, 800 °C, 900 °C, 1000 °C to synthesized different N structure on the surface. The resulting electrode was denoted as N-CNWs/CC-600, N-CNWs/CC-700, N-CNWs/CC-800, N-CNWs/CC-900, N-CNWs/CC-1000, respectively. In preparation of PANI nanowires on carbon cloth, we tested three acidic solutions including HClO_4_, H_2_SO_4_ and HCl for polymerization of PANI. Results indicate that the PANI nanowires deposited on the surface of carbon cloth in HClO_4_ are more uniform than H_2_SO_4_ or HCl, which may be due the better acidity of HClO_4_. Therefore, we selected HClO_4_ to prepare the CNWs/CC. The as-prepared materials were characterized by field emitted scanning electron microscopy (FESEM, JSM-7800F, Japan), Raman spectroscopy (Raman, LabRam HR 800, USA), X-ray photoelectron spectroscopy (XPS, 250Xi, USA), and Micromeritics ASAP 2020. The electroactive surface areas of different electrodes were tested in solution of 50 mM K_3_[Fe(CN)_6_] and calculated according to the Randles-Sevcik formula (Eq. (1))^37^.

1 $$Ip = 2.69 \times 10^5 \times AD^{1/2}n^{3/2}\gamma ^{1/2}C$$

where *A* is the electroactive surface area of the electrode (cm^2^), *D* is the diffusion coefficient of the molecule in solution (cm^2^·s^−1^), *n* stands for electron transfer number, γ is the scan rate and C stands for the concentration of the probe molecule (mol cm^−3^).

### DPV and CV analysis for FMN adsorption ability

Differential pulse voltammetry (DPV) and cyclic voltammograms (CVs) experiments were performed by using CHI 760E electrochemical working station (CHI Instrument, CHI760E, China) in a three-electrode cell that consisted of a working electrode, a saturated calomel electrode (SCE), and a titanium plate counter electrode and conducted in 0.1 mol L^−1^ phosphate buffer solution containing Flavin mononucleotide (FMN, 2 μmol L^−1^). CVs of different N-CNWs/CC electrodes were measured in FMN phosphate buffer solution and test once every four hours interval, and after the peak current is stable, placed these FMN-immobilized electrodes in fresh plain phosphate buffer solution to measure their CVs again. UV spectrophotometer was used to monitor the changes of FMN in solutions after electrochemistry test immediately. All tests were conducted at room temperature.

### MFC setup and operation

Classic H-shaped MFCs were constructed with two 120 mL glass flasks. A Nafion 117 membrane was clamped in a 3.5 cm diameter tubular (8 cm in length) junction to separate the anodic chamber and cathodic chamber. The anode and cathode were placed in the tubular junction close to the membrane to get a shortest distance. The *S. putrefaciens* CN32 in anodic chamber was obtained from Southwest University (P.R.China), and this microorganism could apply lactate as the sole carbon source and generate acetate as the product^11,38^. The bacterial culture was executed according to our previous works^17,19^. In detail, a single clone of *S. putrefaciens* CN32 was cultivated in 5 mL of Luria-Bertani (LB) broth medium (10 g L^−1^ peptone, 5 g L^−1^ yeast extract and 10 g L^−1^ NaCl) overnight, then 1 mL aliquot of bacterial culture suspension was inoculated in 100 mL of fresh LB and incubated with shaking at 30 °C until the optical density at 600 nm (OD600) reached about 1.3. The cell pellets were harvested by centrifugation at 6000 × *g* for 5 min, and then resuspended in 100 mL M9 buffer (Na_2_HPO_4_, 6 g L^−1^, KH_2_PO_4_, 3 g L^−1^, NaCl, 0.5 g L^−1^, NH_4_Cl, 1 g L^−1^, MgSO_4_, 1 mM, CaCl_2_, 0.1 mM), which supplemented with 18 mM lactate as electron donor. The resulted cell suspension was transferred into the anodic chamber of the MFC and purged with nitrogen gas for 30 min to remove the dissolved oxygen. The catholyte was 50 mM K_3_Fe(CN)_6_ solution with 100 mM phosphate buffer (PBS), and carbon brush used as the cathode. The MFCs were operated at room temperature with an external loading resistance (*R*) of 1500 Ω, and the output voltage (*V*) was recorded by a digital multimeter. At the steady-state of the MFC, the polarization and power density curves were obtained by measuring the stable voltage generated at various external loading resistances (200–80,000 Ω). Current was calculated using *I* = *V*/*R*, where *I* was the current, *V* the measured voltage, and *R* the applied external resistance. Power (*P*) was calculated using *P* = *V* × *I*. Both current density and power density were normalized to the anode projected surface area

### Biofilm imaging with FESEM

After MFCs dissembled, small piece of MFCs anode was cut off and the discharged bacteria absorbed anodes were immersed in 4% Polyoxymethylene for 12 h to immobilize bacteria on the electrode surfaces and then sequentially dehydrated with ethanol (30, 40, 50, 60, 70, 80, 90, and 100%) and dried in vacuum at room temperature over-night. Such pretreated biofilms were imaged by FESEM.

### Theoretical calculation

The calculations were based on spin-polarized DFT using PAW methods, as implemented in the VASP^39^. A plane-wave basis set with a kinetic-energy cut-off of 400 eV was used to expand the wave function of valence electrons. The generalized gradient approximation (GGA) with the Perdew-Burke-Ernzerhof (PBE) functional was used for describing the exchange-correlation interactions^40^. 20 Å vacuum space were set to prevent the interaction between two layers. The Brillouin-zone integration was sampled with 3 × 3 × 1 k-points Monkhorst-Pack mesh. The structural relaxations were performed by computing the Hellmann–Feynman forces within the total energy and force convergences of 10^−4^ eV and 0.01 eV/Å, respectively.

## Supplementary information

Supplementary Information

## Data Availability

Authors can confirm that all relevant data are included in the paper and its supplementary information files.
