# Supplementary material for: Nitrogen doping to atomically match reaction sites in microbial fuel cells
Source: Commun Chem. 2020 Jun 1;3:68. doi: 10.1038/s42004-020-0316-z (PMC9814380; doi:10.1038/s42004-020-0316-z)
Supplement: Supplementary file 1 — Supplementary Information [file 42004_2020_316_MOESM1_ESM.pdf]

# Supplementary Information

# Nitrogen doping to atomically match reaction sites in microbial fuel cells

Xiaoshuai Wu [1], Yan Qiao [2,3], Chunxian Guo [1], Zhuanzhuan Shi [1], Chang Ming Li [1,2,3,4*]

[1] Institute of Materials Science and Devices, Suzhou University of Science and Technology, Suzhou 215011, China

[2] Institute for Clean Energy and Advanced Materials, Faculty of Materials and Energy, Southwest University, Chongqing 400715, China

[3] Chongqing Key Laboratory for Advanced Materials and Technologies of Clean Energies, Chongqing 400715, P.R. China

[4] Institute for Advanced Cross-field Science and College of Life Science, Qingdao University, Qingdao 266071, P.R. China

*Corresponding author. Tel/Fax: +86-*023*-68254842; E-mail: ecmli@swu.edu.cn

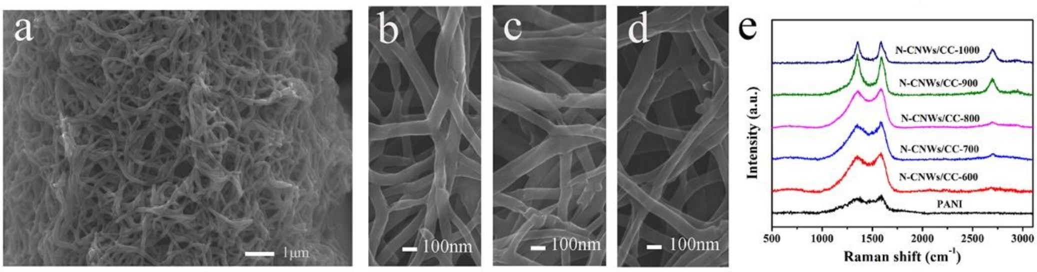


**Supplementary Figure 1**. FESEM micrographs of PANI nanowires without any treatment (a), N-CNWs/CC-700 (b), N-CNWs/CC-800 (c) and N-CNWs/CC-1000 (d). (e) Raman spectra of different N-CNWs/CC.

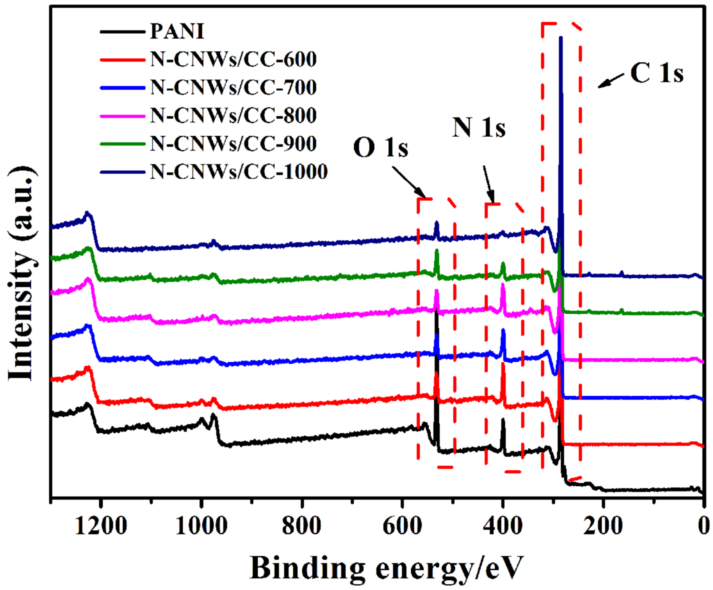


**Supplementary Figure 2.** XPS survey spectra of PANI and different N-CNWs.

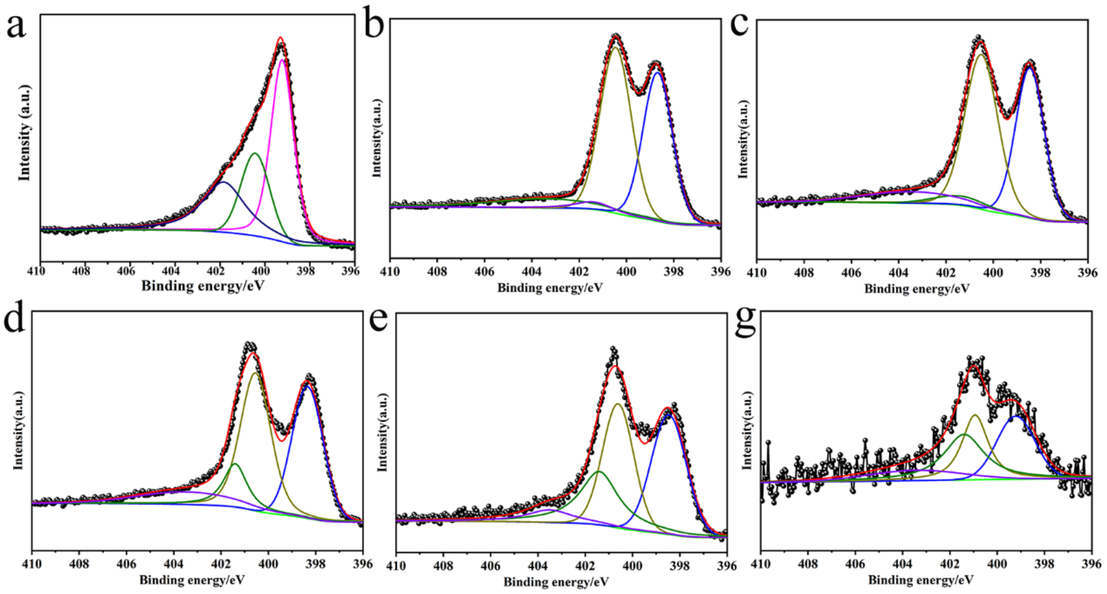


**Supplementary Figure 3.** XPS N1s spectrum of PANI nanowire and N-CNWs (a: PANI nanowire, b: N-CNWs/CC-600, c: N-CNWs/CC-700, d: N-CNWs/CC-800, e: N-CNWs/CC-900, f: N-CNWs/CC-1000).

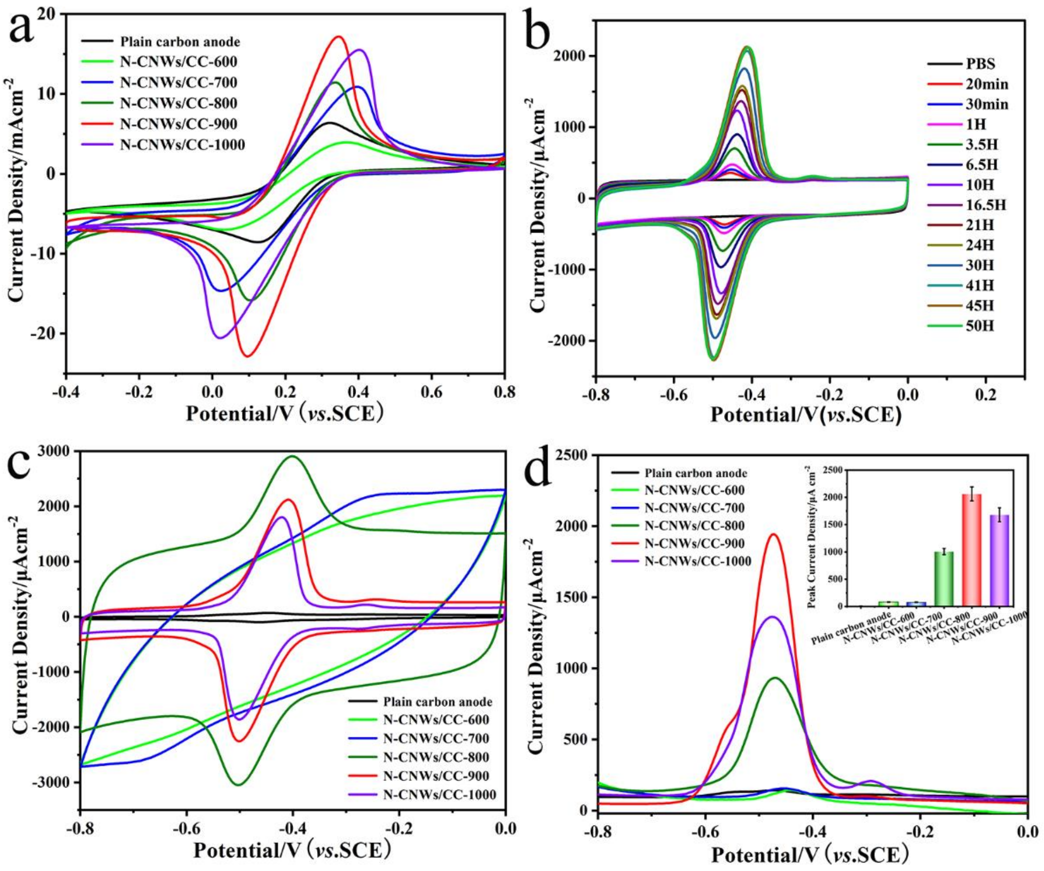


**Supplementary Figure 4.** (a) CVs of N-CNWs electrodes in 50 mM potassium ferricyanide solution. (b) CVs of N-CNWs/CC-900 in 2 μM FMN solution change with time. CVs (c) and DPV (d: the insert is peak current density histogram of different N-CNWs) of FMN spontaneously adsorbing onto N-CNWs electrodes from a 2 μM FMN solution in 0.1M phosphate buffer (PBS) at PH=7.4 over 50h. Error bars represent one standard deviation.

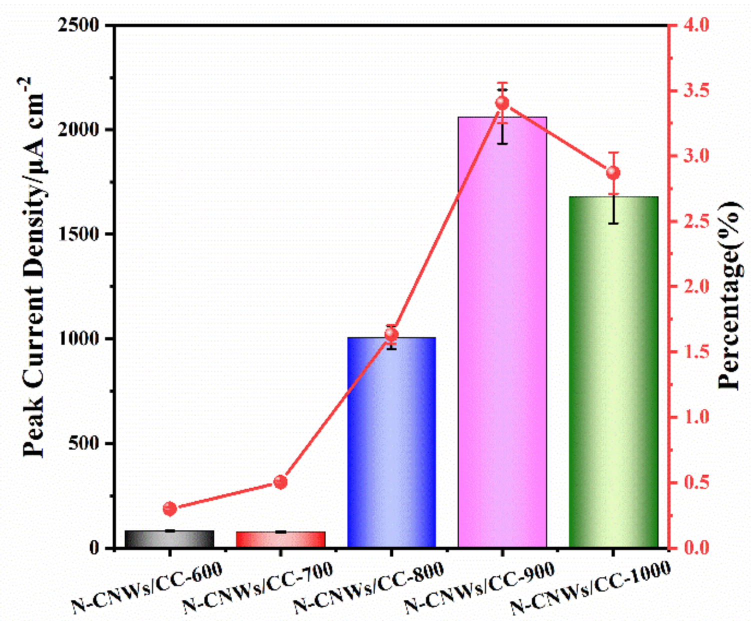


**Supplementary Figure 5.** The peak current and the ratio of Quaternary N over oxidized N of differently N- CNWs/CC electrodes. Error bars represent one standard deviation.

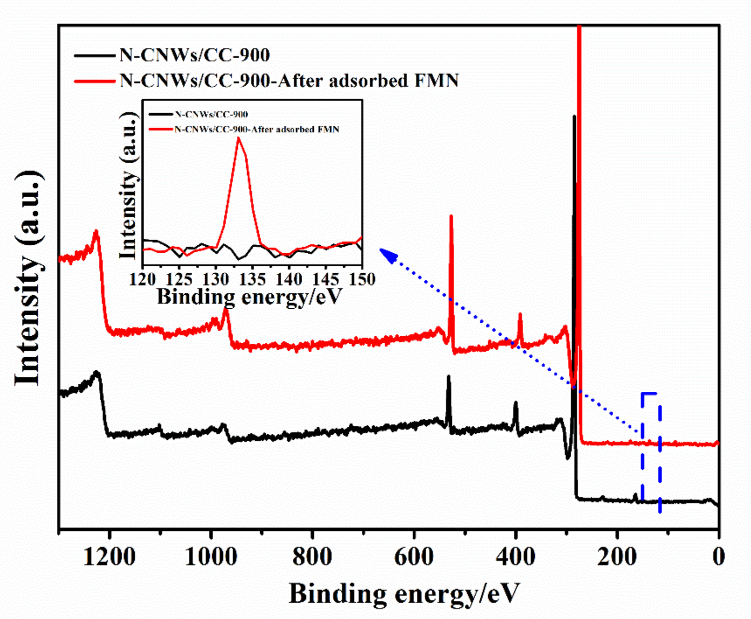


**Supplementary Figure 6.** Total XPS spectra (the insert is P2p spectra) of N-CNWs/CC-900 before and after absorbed FMN.

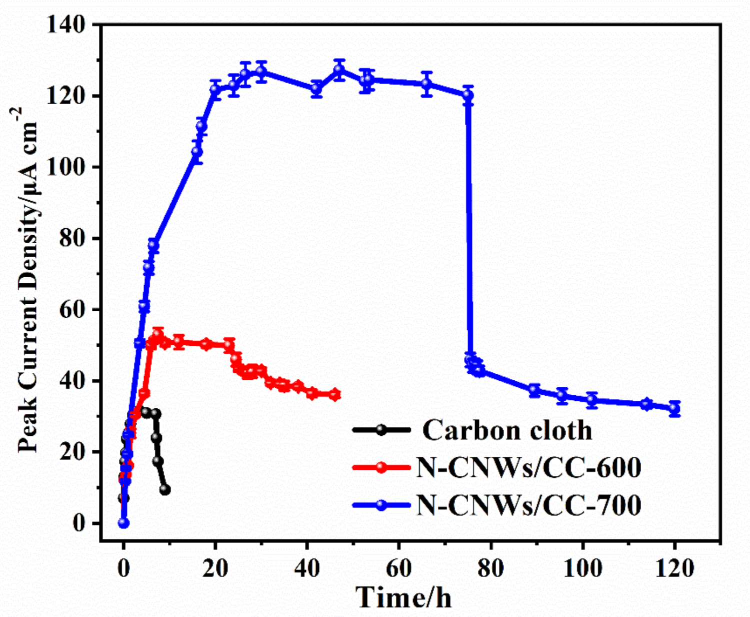


**Supplementary Figure 7.** Peak current density against time of carbon cloth, N-CNWs/CC-600 and N-CNWs/CC-700 in 2 μM FMN solution with 0.1M phosphate buffer (PBS) at PH=7.4. Error bars represent one standard deviation.

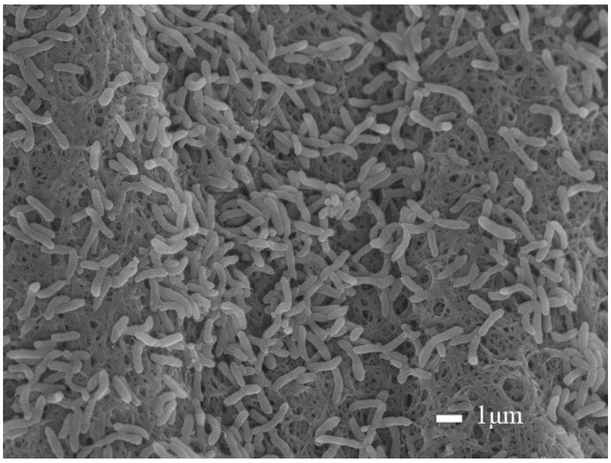


**Supplementary Figure 8.** FESEM micrographs of *S. putrefaciens* cells adhered on the surface of FMN-immobilized electrode.

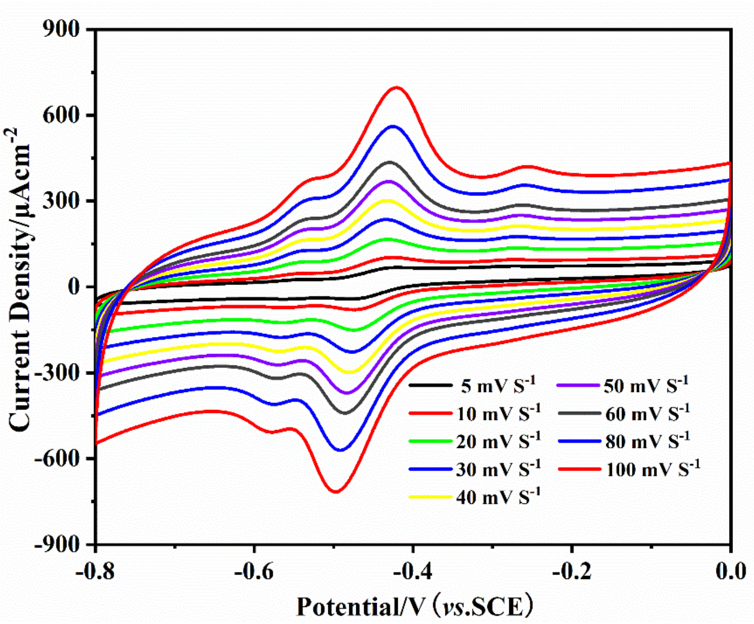


**Supplementary Figure 9.** CV curves at different scan rate of FMN-immobilized electrode measured in an anaerobic of *S. putrefaciens* CN32 suspension with 18 mmol $L^{-1}$ lactate medium.

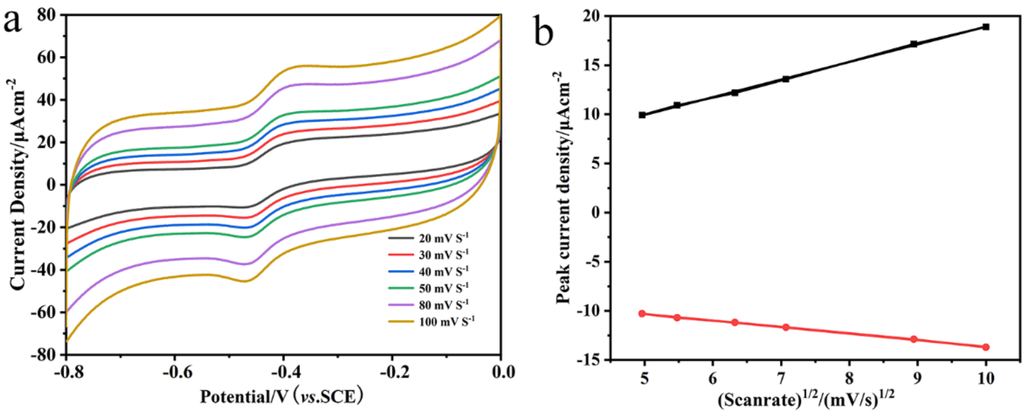


**Supplementary Figure 10.** Peak current density and CV curves at different scan rate of the plain carbon electrode measured in an anaerobic of *S. putrefaciens* CN32 suspension with 18 mmol $L^{-1}$ lactate medium.

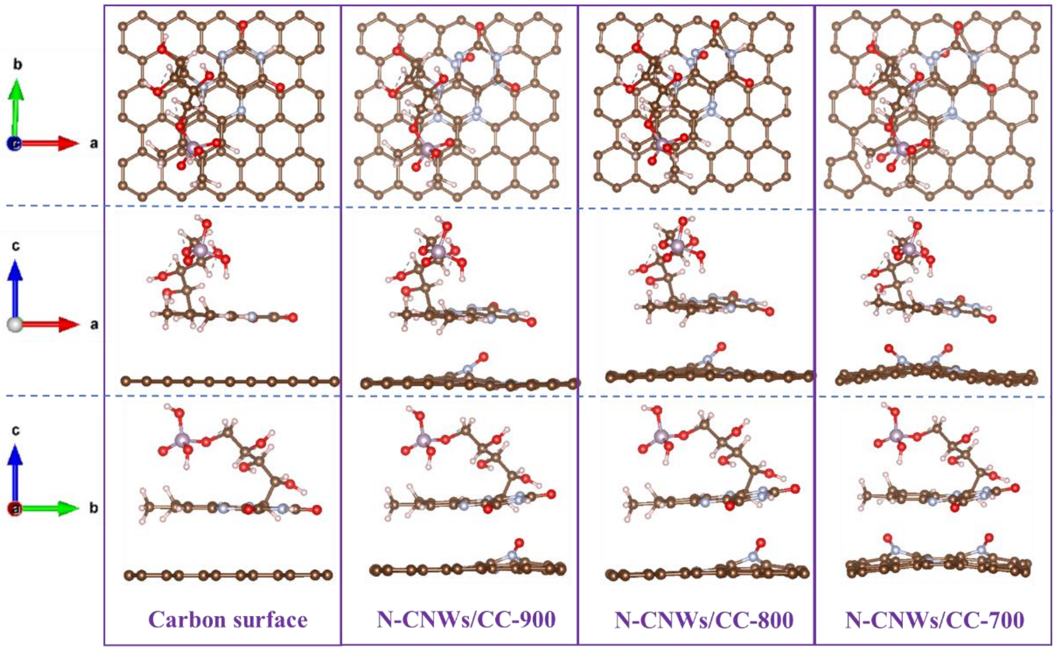


**Supplementary Figure 11.** Optimized structures of FMN adsorbed on carbon surface, N-CNWs/CC-900, N-CNWs/CC-800 and N-CNWs/CC-700,.

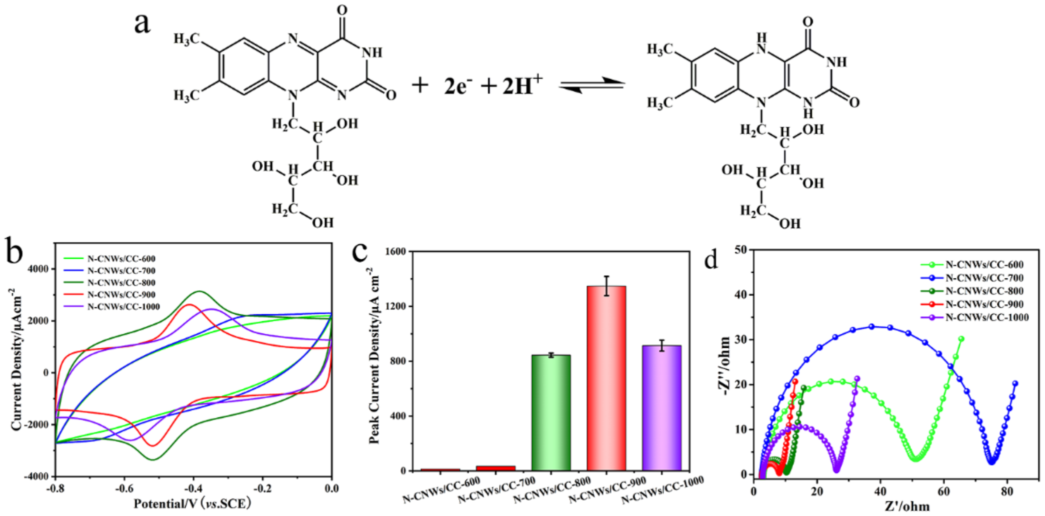


**Supplementary Figure 12.** (a): The molecular structure and the two-electron redox reaction equations of RF. CVs (b), peak current histogram image (c) and Nyquist plots (d) of RF spontaneously adsorbing onto N-CNWs/CC electrodes from a 2 μM RF solution in 0.1M phosphate buffer (PBS) at PH=7.4 over 50h. Error bars represent one standard deviation.

| | N-CNWs/CC-700 | N-CNWs/CC-800 | N-CNWs/CC-900 | N-CNWs/CC-1000 |
|---|---|---|---|---|
| **BET surface area ($m^2/g$)** | 143.9 | 148.1 | 167.3 | 170.6 |
| **Pore Size (nm)** | 3.071 | 3.661 | 2.863 | 2.762 |
| **Water contact angle (°)** | 101.8 | 102.5 | 103.6 | 106.9 |
| **Electron conductivity (Ω)** | 13.83 | 8.376 | 6.995 | 13.07 |
| **Electroactive surface area ($cm^2/g$)** | 371.9 | 426.1 | 601.3 | 557.58 |

**Supplementary Table 1** Summary of BET surface area, pore size, water contact angle, and electron conductivity of the nanowires after carbonized at different temperatures.

| Sample | Content of nitrogen atoms | Pyridine N 398.5±0.3eV | Pyridine or Pyrrole N 400.5±0.3eV | Quaternary N 401.2±0.3eV | Oxidized N 402.9±0.3eV | Ratio of Quaternary N and Oxidized N | O1s peak connect with nitrogen 533.3±0.3eV |
|---|---|---|---|---|---|---|---|
| **N-CNWs-600** | 10.94% | 4.25% | 5.02% | 0.37% | 1.3% | **0.28** | 1.8% |
| **N-CNWs-700** | 10.345% | 4.03% | 4.85% | 0.445% | 1.02% | **0.437** | 1.5% |
| **N-CNWs-800** | 8.19% | 2.94% | 3.36% | 1.01% | 0.88% | **1.15** | 2.09% |
| **N-CNWs-900** | 7.8% | 2.55% | 2.52% | 1.9% | 0.53% | **3.55** | 2.1% |
| **N-CNWs-1000** | 0.624% | 0.18% | 0.17% | 0.2% | 0.074% | **2.74** | 1.5% |

**Supplementary Table 2** Distribution of N species obtained from the deconvolution of the N1s peak of N-CNWs.

| Electrode | Inoculum | Substrate | Device type | Performance | Ref. |
|---|---|---|---|---|---|
| FMN-immobilized atomic matched nitrogen doped anode | *S. putrefaciens CN32* | Lactate medium | Dual-chamber MFC | 2102.88 mW $m^{-2}$ | This work |
| graphene-containing foam | *S. putrefaciens* | Lactate medium | Dual-chamber MFC | 786 mW $m^{-2}$ | 1 |
| Carbon nanotubes and polyaniline (PANI) on microporous graphite felt | *S. putrefaciens* | Acetate medium | Dual-chamber MFC | 308 mW $m^{-2}$ | 2 |
| PANI networks onto graphene nanoribbons coated carbon paper | S. oneidensis | Lactate medium | Dual-chamber MFC | 856 mW $m^{-2}$ | 3 |
| Graphene aerogel | *S. putrefaciens CN32* | Lysogeny broth (LB) medium | Dual-chamber MFC | 679.7 mW $m^{-2}$ | **4** |
| Graphene foam/PANI | *S. oneidensis* MR-1 | Lactate medium | Dual-chamber MFC | 768 mW $m^{-2}$ | 5 |
| $TiO_2$ nanoscrystal/rGO | *S. putrefaciens* CN32 | LB medium | Dual-chamber MFC | 540 mW $m^{-2}$ | **6** |
| Graphene/amorphous $TiO_2$ | *S. oneidensis* | Lactate medium | Dual-chamber MFC | 1060 mW $m^{-2}$ | **7** |
| N-doping graphene aerogel (N-GA) | *S. oneidensis* MR-1 | Trypticase soy broth | Dual-chamber MFC | 1990.8±106.1 mW $m^{-2}$ | 8 |
| Macroporous graphitic carbon foam polydopamine (PDA) | *S. putrefaciens* | Lactate medium | Dual-chamber MFC | 1735 mW $m^{-2}$ | 9 |
| Mo2C) functionalized carbon felt | *S. putrefaciens* CN32 | Lactate medium | Dual-chamber MFC | 1025 mW $m^{-2}$ | 10 |
| Polyaniline hybridized large mesoporous carbon (PANI-LMC) | *S. putrefaciens* CN32 | Lactate medium | Dual-chamber MFC | 1280 mW $m^{-2}$ | 11 |

**Supplementary Table 3** Summary of reported bioelectrode materials for MFCs applications with S. putrefaciens or S. oneidensis as biocatalyst.

| Structure | Carbon surface | N-CNWs-900 | N-CNWs-800 | N-CNWs-700 |
|---|---|---|---|---|
| **$E_{basel}$ (eV)** | -553.713 | -537.158 | -541.884 | -531.586 |
| **$E_{FMN}$ (eV)** | -332.711 | -332.711 | -332.711 | -332.711 |
| **$E_{total}$ (eV)** | -886.431 | -870.129 | -874.683 | -864.356 |
| **$E_{ads}$ (eV)** | -0.007 | -0.260 | -0.088 | -0.059 |

**Supplementary Table 4** Adsorption energy of FMN adsorbed on different electrode interface.

## Supplementary References


1. Yang L, *et al.* Facile Fabrication of Graphene-Containing Foam as a High-Performance Anode for Microbial Fuel Cells. *Chem Eur J* **21**, 10634-10638 (2015).
2. Cui H-F, Du L, Guo P-B, Zhu B, Luong JHT. Controlled modification of carbon nanotubes and polyaniline on macroporous graphite felt for high-performance microbial fuel cell anode. *J Power Sources* **283**, 46-53 (2015).
3. Zhao C, *et al.* Polyaniline networks grown on graphene nanoribbons-coated carbon paper with a synergistic effect for high-performance microbial fuel cells. *J Mater Chem A* **1**, 12587-12594 (2013).
4. Qiao Y, Wen G-Y, Wu X-S, Zou L. L-Cysteine tailored porous graphene aerogel for enhanced power generation in microbial fuel cells. *RSC Adv* **5**, 58921-58927 (2015).
5. Yong Y-C, Dong X-C, Chan-Park MB, Song H, Chen P. Macroporous and Monolithic Anode Based on Polyaniline Hybridized Three-Dimensional Graphene for High-Performance Microbial Fuel Cells. *ACS Nano* **6**, 2394-2400 (2012).
6. Zou L, Qiao Y, Wu X-S, Ma C-X, Li X, Li CM. Synergistic effect of titanium dioxide nanocrystal/reduced graphene oxide hybrid on enhancement of microbial electrocatalysis. *J Power Sources* **276**, 208-214 (2015).
7. Zhao C-e, Wang W-J, Sun D, Wang X, Zhang J-R, Zhu J-J. Nanostructured Graphene/TiO2 Hybrids as High-Performance Anodes for Microbial Fuel Cells. *Chem Eur J* **20**, 7091-7097 (2014).
8. Yang Y, *et al.* Boosting Power Density of Microbial Fuel Cells with 3D Nitrogen-Doped Graphene Aerogel Electrode. *Adv Sci* **3**, 1600097 (2016).

9. Jiang H, Yang L, Deng W, Tan Y, Xie Q. Macroporous graphitic carbon foam decorated with polydopamine as a high-performance anode for microbial fuel cell. *J Power Sources* **363**, 27-33 (2017).

10. Zou L, Lu Z, Huang Y, Long Z-e, Qiao Y. Nanoporous Mo2C functionalized 3D carbon architecture anode for boosting flavins mediated interfacial bioelectrocatalysis in microbial fuel cells. *J Power Sources* **359**, 549-555 (2017).

11. Zou L, Qiao Y, Zhong C, Li CM. Enabling fast electron transfer through both bacterial outer-membrane redox centers and endogenous electron mediators by polyaniline hybridized large-mesoporous carbon anode for high-performance microbial fuel cells. *Electrochim Acta* **229**, 31-38 (2017).
